# Trimedazidine alleviates pulmonary artery banding-induced acute right heart dysfunction and activates PRAS40 in rats

**DOI:** 10.18632/oncotarget.20752

**Published:** 2017-09-08

**Authors:** Yunshan Cao, Jiyang Song, Shutong Shen, Heling Fu, Xiang Li, Ying Xu, Aqian Wang, Xinli Li, Min Zhang

**Affiliations:** ^1^ Department of Cardiology, Gansu Provincial Hospital, Lanzhou 730000, China; ^2^ Department of Heart Failure, Shanghai East Hospital, Tongji University School of Medicine, Research Center for Translational Medicine, Shanghai 200120, China; ^3^ Department of Cardiology, The First Affiliated Hospital with Nanjing Medical University, Nanjing 210029, China; ^4^ Animal Core Facility, Nanjing Medical University, Nanjing 210029, China; ^5^ Department of Intensive Care, Minhang Hospital, Fudan University, Shanghai 201100, China; ^6^ Intensive Care Unit, Nanjing Drum Tower Hospital, The Affiliated Hospital of Nanjing University Medical School, Nanjing 210008, China; ^7^ Department of Pathology, Gansu Provincial Hospital, Lanzhou 730000, China

**Keywords:** acute right dysfunction, trimedazidine, PRAS40, pulmonary artery banding

## Abstract

The molecular mechanism underlying acute right heart failure (RHF) is poorly understood. We used pulmonary artery banding (PAB) to induce acute RHF characterized by a rapid rise of right ventricular pressure, and then a decrease in right ventricular pressure along with a decrease in blood pressure right after banding. We found higher brain natriuretic peptide (BNP) and beta-myosin heavy chain (βMHC) levels and lower alpha-myosin heavy chain (αMHC) levels in RHF rats than sham-operated rats. Hemodynamic indexes in rats with acute RHF were slightly improved by trimedazidine TMZ, a key inhibitor of fatty acid (FA) oxidation. TMZ also reversed downregulation of peroxisome proliferator-activated receptor gamma coactivator 1-beta (PGC-1β) and peroxisome proliferator-activated receptor alpha (PPARα) by PAB and up-regulates peroxisome proliferator-activated receptor gamma coactivator 1-alpha (PGC-1α), peroxisome proliferator-activated receptor delta (PPARδ) and pyruvate dehydrogenase kinase isoform 4 (PDK4). In addition, TMZ reversed upregulation of phosphorylated Akt by PAB and increased phosphorylated proline-rich Akt-substrate 40 (PRAS40). Autophagy and apoptosis were not modified by PAB or TMZ. An acute RHF model was established in rats through 70% constriction of the pulmonary artery. TMZ treatment alleviated PAB-induced acute RHF by activating PRAS40 and upregulatingPGC-1α, PGC-1β, PPARα, PPARδ, and PDK4.

## INTRODUCTION

Acute right heart failure (RHF) is a common but rarely studied disease that might be caused by rapidly increased right ventricle (RV) afterload during acute pulmonary embolism, hypoxic pulmonary vasoconstriction, or after cardiac transplantation or prolonged cardiopulmonary bypass. In these conditions, acute RHF is a major cause of morbidity and mortality. However, according to the Working Group on Cellular and Molecular Mechanisms of Right Heart Failure of the National Heart, Lung and Blood Institute, there is “a paucity of basic knowledge at all levels about the RV's normal and pathological function” [1]. Therefore, the molecular mechanisms of RV dysfunction should be investigated.

The protein kinase B, also known as Akt, signaling pathway is a factor in cardiac hypertrophy, remodeling, and cardiomyocyte proliferation in the left ventricle (LV) [2, 3]. Activated Akt protects cardiomyocytes from apoptosis induced by ischemia-reperfusion injury *in vivo* [4]. Exercise can activate Akt, which was observed after pressure and volume overload in rabbit models [5, 6]. Cardiac-specific constitutively active Akt prevented cardiac dilatation and sudden death in an animal model [7]. However, few studies have been conducted on Akt signaling in the right heart. The RV is embryologically, structurally, and physiologically different from the LV [8, 9]. Akt signaling is an important factor in the development of the RV [10]. Our previous study found that phosphorylated Akt strongly expressed in the threonine 308 site but not in the serine 473 site in the RV compared with the LV, and proline-rich Akt-substrate 40 (PRAS40), an indicator of Akt activity, was significantly increased in its phosphorylated form but total PRAS40 was not increased in the RV compared with the LV [11]. In PAB-induced acute RHF, the effects of Akt signaling are still unknown.

In normal adults, blood flow in the right coronary artery mostly occurs during systole, whereas blood flow in the left coronary artery mostly occurs during diastole [12]. Therefore, pressure overload can predispose the RV to dysfunction and subendocardial ischemia compared with the LV [12, 13]. Studies demonstrated that coronary perfusion occurs primarily during diastole in patients with PH [14] and capillary density was reduced in several PH models [15–17], which caused a mismatch between decreased coronary blood supply and increased oxygen demand in the RV. Prostacyclin therapy in rats with PH was reported to improve both capillary density and survival [15]. Oxygen radical scavengers were also increased in rats with MCT-induced chronic PH [18]. In addition, metabolic shifts from fatty acid oxidation (FAO) to glycolysis were observed in the chronic pressure-loaded RV [19] and in MCT-induced chronic PH [20, 21]. Studies have suggested that reducing FAO and increasing glucose oxidation (GO) are promising treatments in heart failure patients [22]. Trimetazidine (TMZ) is an anti-anginal agent and can attenuate cardiac FAO and shifts metabolism to GO via the Randle cycle by selectively inhibiting long-chain 3-ketoacyl CoA thiolase (3-KAT) [23]. TMZ can relieve mild RV dysfunction caused by long-term PAB [24]. However, in the acute RHF model, whether changes occur in metabolic gene profiles and whether TMZ has some protective effects in right heart function are still unknown.

We developed a PAB-induced acute right heart model evaluated by hemodynamic measurement. We found that Akt activity was increased and peroxisome proliferator-activated receptor gamma coactivator 1-beta (PGC-1β) and peroxisome proliferator-activated receptor alpha (PPARα) were decreased in rats with PAB compared with rats in the sham group. TMZ pre-treatment before PAB can alleviate PAB-induced acute right heart dysfunction and activate PRAS40 as well as upregulate peroxisome proliferator-activated receptor gamma coactivator 1-alpha (PGC-1α), PGC-1β, PPARα, peroxisome proliferator-activated receptor delta (PPARδ) and pyruvate dehydrogenase lipoamide kinase isozyme 4 (PDK4). TMZ pre-treatment might be an alternative for patients with acute RHF caused by rapidly increased afterload such as pulmonary hypertension and pulmonary embolism.

## RESULTS

### Development of acute right heart failure in rats

Supplementary Figures 1A, 1E, and 1F show that less than 71% constriction degree caused a rapid increase in maximum right ventricular pressure (RVP max), and then a persistent high level lasting at least 30 minutes. More than 70% constriction degree caused a rapid increase, and then a decrease in RVP max. Neither blood pressure (BP) nor heart rate (HR) were changed in the less than 71% constriction degree group but were significantly decreased in the more than 70% constriction degree group compared with the control group (Supplementary Figures 1A, 1B, 1C, and 1D). The positive rate of rise of right ventricle pressure (+dP/dT) decreased, whereas the negative rate of rise right ventricle pressure (−dP/dT) significantly increased compared with control (Supplementary Figures 1G and 1H).

An acute RHF model in rats was established by PAB of more than 70% constriction degree.

### Short-term PAB induced increases in pathological markers of the RV that were reversed by TMZ treatment in rats

**Figure 1:**
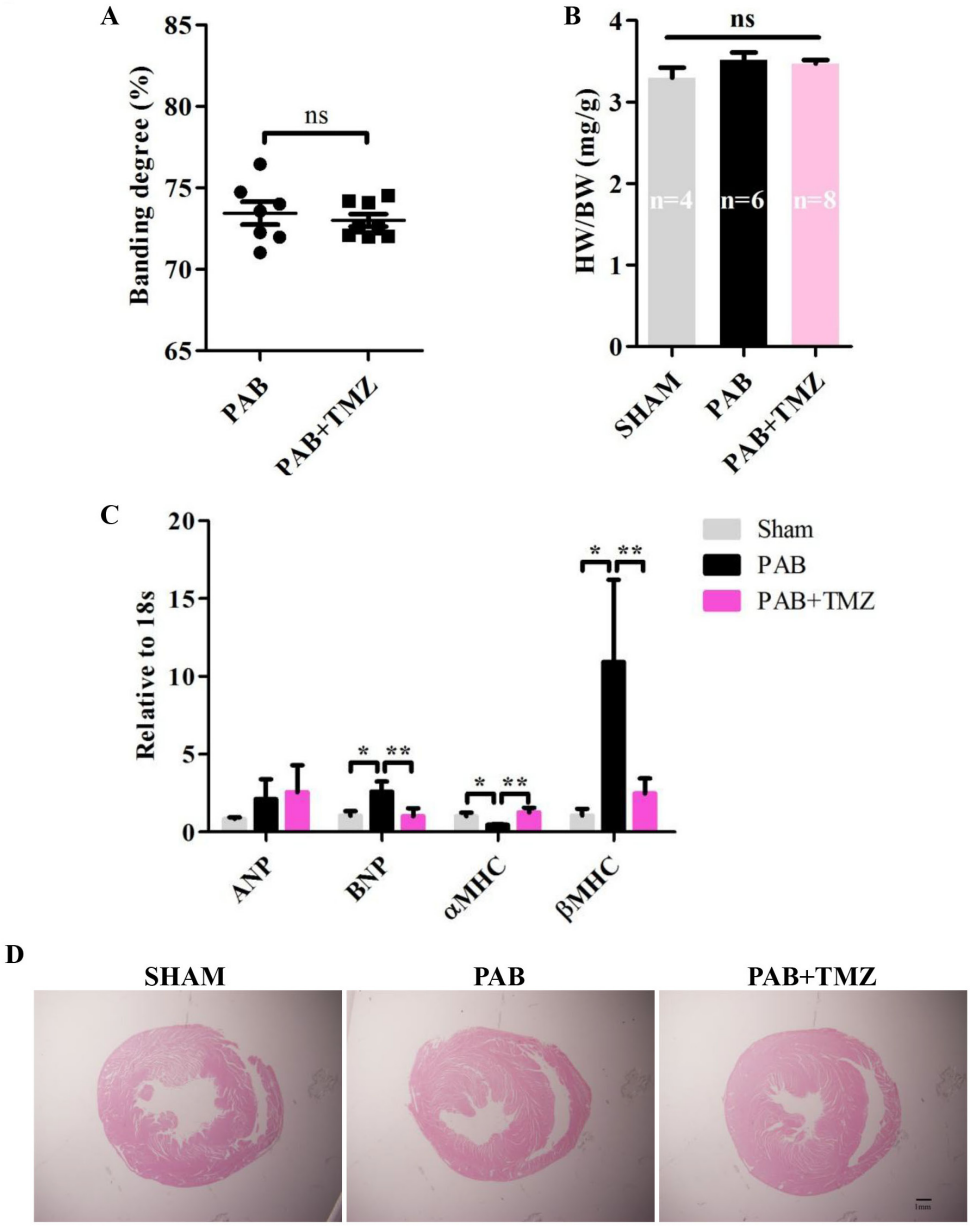
PAB increased mRNA levels of RV pathologic markers and TMZ reversed effects in rats **(A)** Statistical analysis of constriction degree (%) in the PAB and PAB+TMZ groups. **(B)** Statistical analysis of the ratio of heart weight to body weight in the sham, PAB, and PAB+TMZ groups. **(C)** mRNA levels of ANP, BNP, αMHC, and βMHC in the sham, PAB and PAB+TMZ groups. **(D)** Representative H&E staining of histological section in the sham, PAB, and PAB+TMZ groups (Scale bar = 1.0 mm). Abbreviations: PAB, pulmonary artery banding; TMZ, trimedazidine; ANP, atrial natriuretic peptide; BNP, brain natriuretic peptide; αMHC, alpha myosin heavy chain; βMHC, beta myosin heavy chain; H&E, hematoxylin and eosin. ^*^P < 0.05, ^**^P < 0.01, n = 4-8 in each group.

Figure 1A shows no significant differences in constriction degree between the PAB group and the PAB+TMZ group. Among sham, PAB, and PAB+TMZ groups, the ratio of heart weight to body weight had no significant differences (Figure 1B).

Compared with the sham group, brain natriuretic peptide (BNP) and beta myosin heavy chain (βMHC) increased in the PAB group 30 minutes after banding, but atria natriuretic peptide (ANP) did not increase (Figure 1C). Conversely, alpha myosin heavy chain (αMHC) decreased significantly in the PAB group compared with the sham group (Figure 1C). TMZ treatment decreased BNP and βMHC levels and increased αMHC levels but had no effect on ANP levels compared with the PAB group (Figure 1C).

Furthermore, compared with the sham group, the RV dilated in the PAB group, and TMZ treatment did not change RV size (Figure 1D).

Short-term PAB caused RHF indicated by pathological markers, and TMZ alleviated the damage of RHF caused by PAB.

### Short-term PAB caused acute RV failure that was improved by TMZ treatment in rats

**Figure 2:**
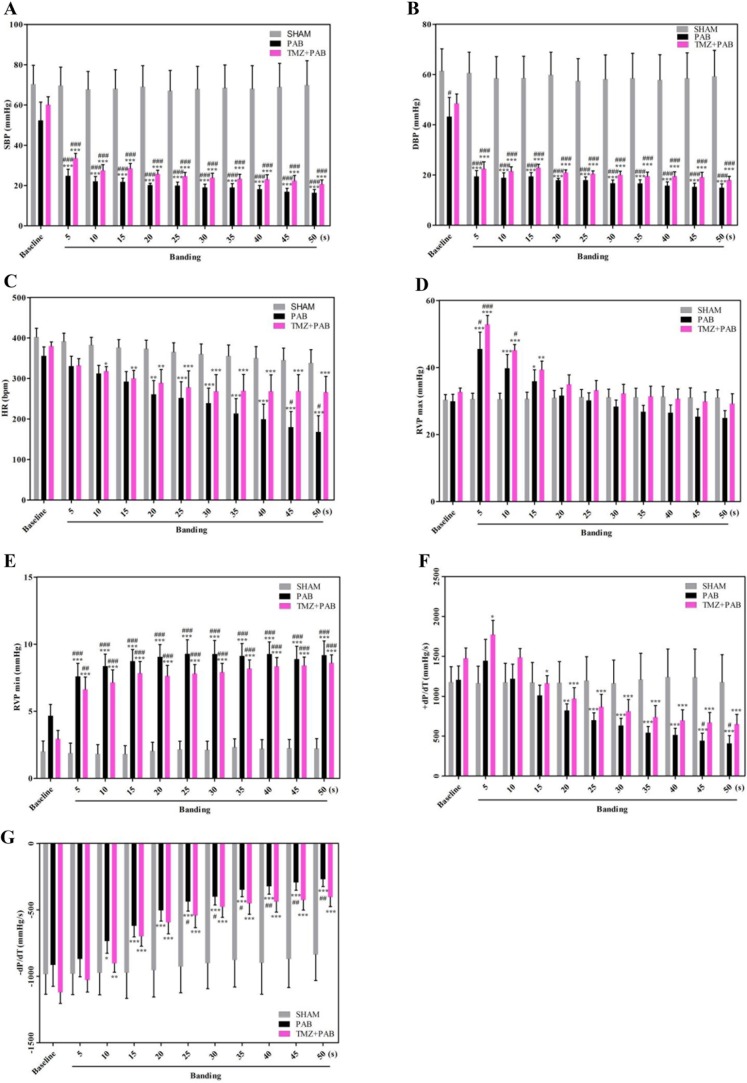
Comparison of hemodynamic parameters among the sham, PAB, and PAB+TMZ groups in rat **(A-G)** Statistical analysis of hemodynamic parameters (SBP, DBP, HR, RVP max, RVP min, +dP/dT, and –dP/dT). Abbreviations: PAB, pulmonary artery banding; TMZ, trimedazidine; SBP, systolic blood pressure; DBP, diastolic blood pressure; HR, heart rate; RVP max, maximum right ventricle pressure; RVP min, minim right ventricle pressure. ^*^P<0.05, ^**^P < 0.01, compared with baseline; ^#^*P* < 0.05, compared with sham group; n = 4-8 in each group.

Figures 2A and 2B show that both systolic blood pressure (SBP) and diastolic blood pressure (DBP) decreased in the PAB group compared with both the sham group and baseline 5 seconds after banding. Baseline SBP was not different among the sham, PAB, and PAB+TMZ groups, whereas DBP was lower in the PAB group than in the sham group at baseline. In the PAB+TMZ group, both SBP and DBP decreased compared with the sham group and baseline 5 seconds after banding, but increased compared with the PAB group (Figure 2A and 2B). Heart rate (HR) decreased in the PAB group compared with baseline 20 seconds after banding and compared with the sham group 45 seconds after banding (Figure 2C). In the PAB+TMZ group, HR decreased compared with baseline 10 seconds after banding and had no significant differences from the sham group. Although there were no significant differences in HR between the PAB+TMZ group and the PAB group, the decline in HR in the PAB+TMZ group was not as much as in the PAB group compared with the sham group (Figure 2C). The RVP max was increased in the PAB group at 5 seconds, 10 seconds, and 15seconds after banding compared with baseline and 5 seconds after banding compared with the sham group (Figure 2D).

Figure 2D shows that after an abrupt increase in RVP max after banding, RVP max gradually decreased. Compared with the sham group and baseline, RVP max increased in the PAB and PAB+TMZ groups at 5 seconds after banding. The RVP max was still higher in the PAB+TMZ group but not in the PAB group compared with the sham group 10 seconds after banding. No significant differences in RVP max occurred among the sham, PAB, and PAB+TMZ groups and in the PAB+TMZ and PAB groups compared with baseline at and after 20 seconds of banding, but the RVP max of the PAB+TMZ group was still higher than that of the PAB group. The minimum RVP (RVP min) was increased in the PAB group and the PAB+TMZ group 5 seconds after banding compared with both baseline and the sham group (Figure 2E). Consistent with the RVP trends, +dP/dT increased rapidly after banding, and then decreased gradually (Figure 2F). Beginning 20 seconds after banding, +dP/dT decreased in the PAB and PAB+TMZ groups compared with baseline (Figure 2F). At 45 seconds and 50 seconds after banding, +dP/dT decreased in the PAB group but not in the PAB+TMZ group compared with the sham group (Figure 2F). −dP/dT increased in the PAB group and the PAB+TMZ group 10 seconds after banding compared with baseline. Compared with the sham group, −dP/dT increased significantly in the PAB group but not in the PAB+TMZ group 25 seconds after banding (Figure 2G). The increased levels of −dP/dT and the decreased levels of +dP/dT were higher in the PAB group than in the PAB+TMZ group (Figure 2F and 2G).

**Figure 3:**
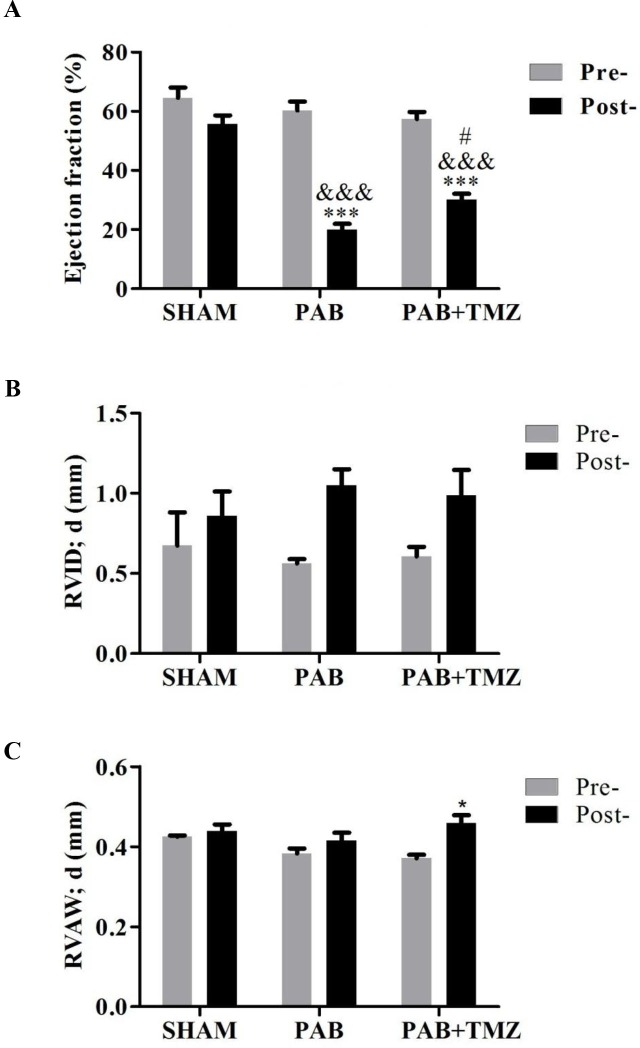
Comparison of right ventricular ejection fraction (EF), RVAWd, and RVIDd among the sham, PAB, and PAB+TMZ groups 24 hours after operation in rats **(A)** Right ventricular ejection fraction; **(B)** RVID; d; **(C)** RVAW; d. Abbreviations: PAB, pulmonary artery banding; TMZ, trimedazidine; RVID; d, right ventricle internal diameter in diastole; RVAW; d, right ventricle anterior wall thickness in diastole; Pre-, pre-PAB; Post-, 24 hours after PAB. ^*^P < 0.05, ^***^P < 0.001, Post- versus Pre-; *^&&&^P* < 0.001, PAB, PAB+TMZ versus sham; ^#^*P* < 0.05, PAB+TMZ versus PAB; n = 3-5 in each group.

Figures 3A, 3B, and 3C show that ejection fraction (EF) of the RV decreased in the PAB group compared with baseline and the sham group, and RV intradiameter in diastole (RVID; d) and RV anterior wall thickness in diastole (RVAW; d) had no significant differences between the PAB group and baseline as well as the PAB group and the sham group. However, RVID; d increased more in post-PAB compared with pre-PAB in the PAB and PAB+TMZ groups than in the sham group. The decline of EF was significantly lower in the PAB+TMZ group after banding compared with the PAB group.

According to hemodynamic parameters, TMZ treatment partially improved PAB-induced acute right heart dysfunction.

### PGC-1β and PPARα were downregulated by short-term PAB, and TMZ treatment upregulated PGC-1α, PGC-1β, PPARα, PPARδ, and PDK4 in rats

**Figure 4:**
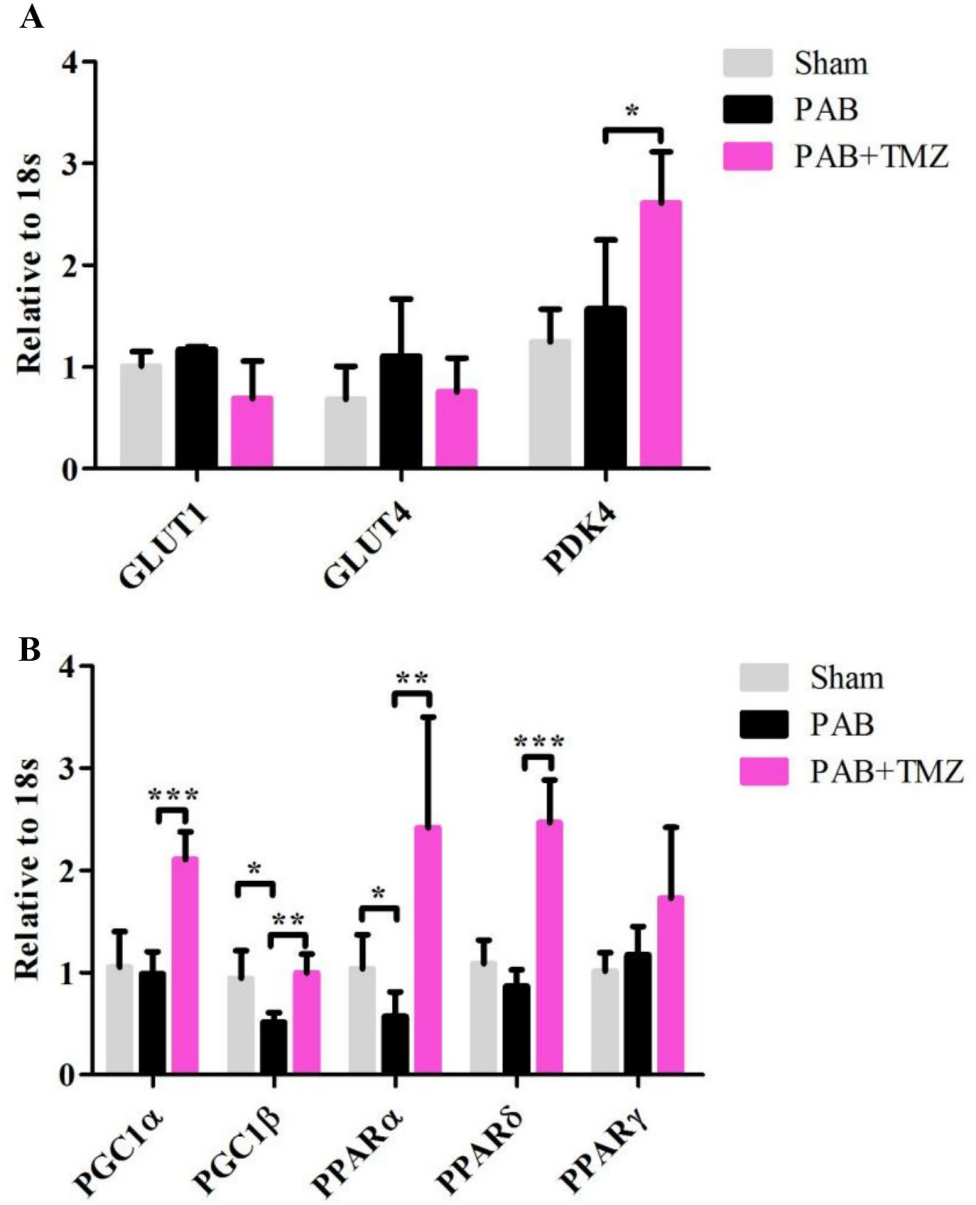
Metabolic gene profile changed after PAB and PAB+TMZ treatment **(A)** Glucose metabolic gene profile (GLUT1, GLUT4, and PDK4) in the sham, PAB, and PAB + TMZ groups. **(B)** Mitochondrial metabolic gene profile (PGC1α and PGC1β) and lipid metabolic gene profile (PPARα, PPARδ, and PPARγ) in the sham, PAB, and PAB + TMZ groups. Abbreviations: PAB, pulmonary artery banding; TMZ, trimedazidine; GLUT1, glucose transporter 1; GLUT4, glucose transporter 4; PDK4, pyruvate dehydrogenase lipoamide kinase isozyme 4; PGC-1α, peroxisome proliferator-activated receptor gamma coactivator 1-alpha; PGC-1β, peroxisome proliferator-activated receptor gamma coactivator 1-beta; PPARα, peroxisome proliferator-activated receptor alpha; PPARδ, peroxisome proliferator-activated receptor delta; PPARγ, peroxisome proliferator-activated receptor gamma. ^*^P < 0.05, ^**^P < 0.01, ^***^P < 0.001, n = 4-8 in each group.

Figure 4A shows no significant differences in PDK4, glucose transporter 1 (GLUT1) and glucose transporter 4 (GLUT4) between the PAB group and baseline as well as the PAB and sham groups. PGC-1β and PPARα were decreased in the PAB group compared with the sham group but not PGC-1α, PPARδ, and peroxisome proliferator-activated receptor gamma (PPARγ) (Figure 4B). Compared with the PAB group, besides PGC-1β and PPARα, TMZ increased PDK4, PGC-1α, and PPARδ (Figure 4A and 4B).

### TMZ treatment increased PRAS40 activity and decreased Akt activity induced by short-term PBA in rats

**Figure 5:**
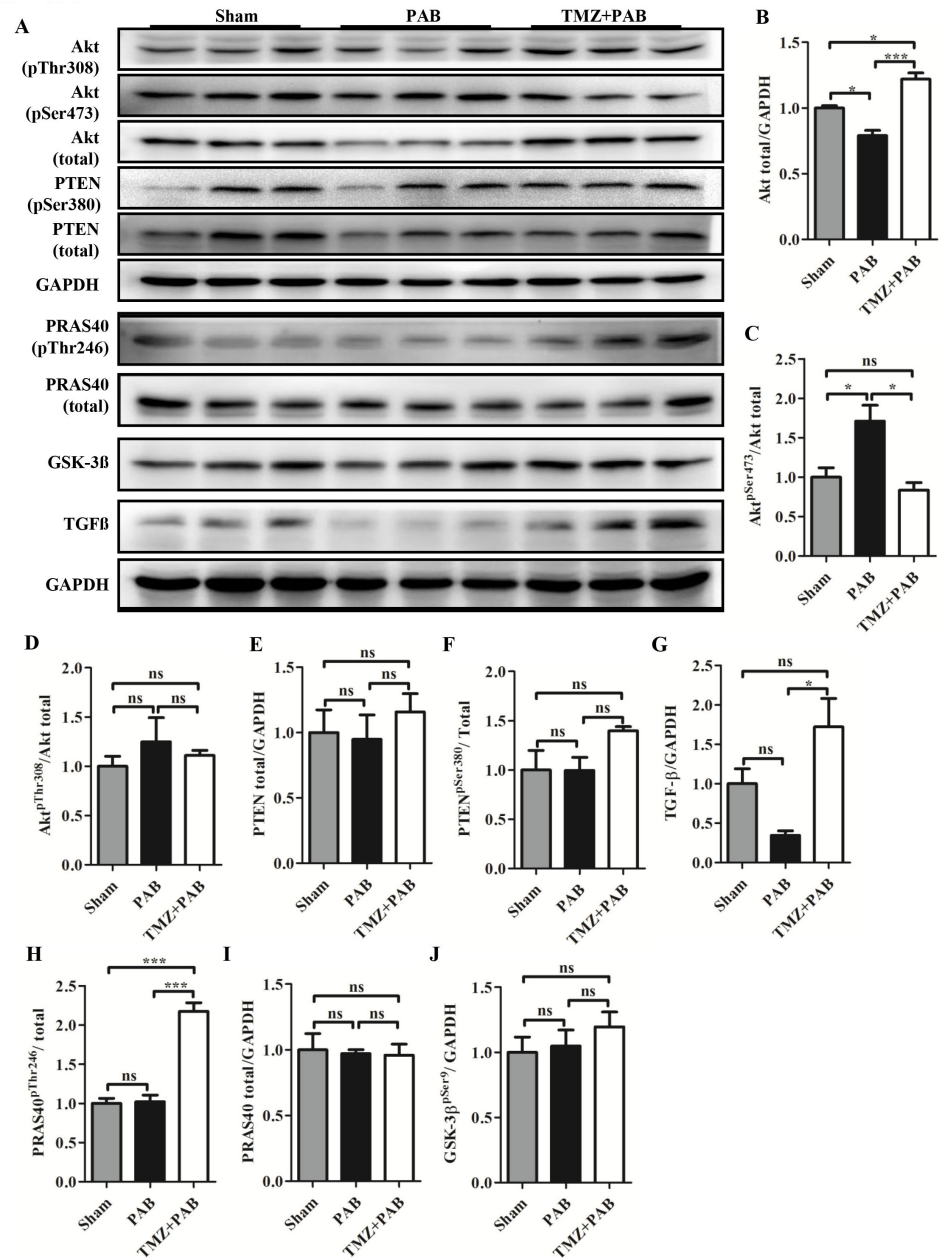
Detection of Akt signaling pathway by Western blot in each group **(A)** Representative WB images. **(B-J)** Statistical analysis of (A). Abbreviations: PAB, pulmonary artery banding; TMZ, trimedazidine; Akt, protein kinase B; PTEN, phosphatase and tensin homolog; TGF-β, transforming growth factor-beta; PRAS40, proline-rich Akt-substrate 40; GSK-3β, glycogen synthase kinase-3 beta; GAPDH, glyceraldehyde 3-phosphate dehydrogenase. ^*^P<0.05, ^**^P<0.01, ^***^P<0.001, n=4-8 in each group.

Figure 5A shows that total Akt was decreased in the PAB group compared with the sham group. Correspondingly, levels of phosphorylated Akt at the serine 473 site was increased but not at the threonine site in the PAB group compared with the sham group (Figures 5A, 5B, 5C, and 5D). Levels of phosphatase and tensin homolog (PTEN), transforming growth factor-beta (TGF-β), PRAS40, and Glycogen synthase kinase-3 beta (GSK-3β) were not different between the PAB and sham groups (Figures 5A and 5E-5J). TMZ treatment increased PRAS40 activity, indicated by increased phosphorylated PRAS40, and inhibited Akt activity, indicated by decreased phosphorylated Akt at the serine 473 site (Figures 5A-5C and 5H). TMZ treatment had no effects on PTEN, GSK-3β, total PRAS40, and phosphorylated Akt at the therine 308 site. Compared with the sham and PAB groups, TMZ treatment increased TGF-β level (Figure 5A and 5G).

### The autophagy and the apoptosis pathways were not altered by short-term PAB and TMZ treatment in rats

**Figure 6:**
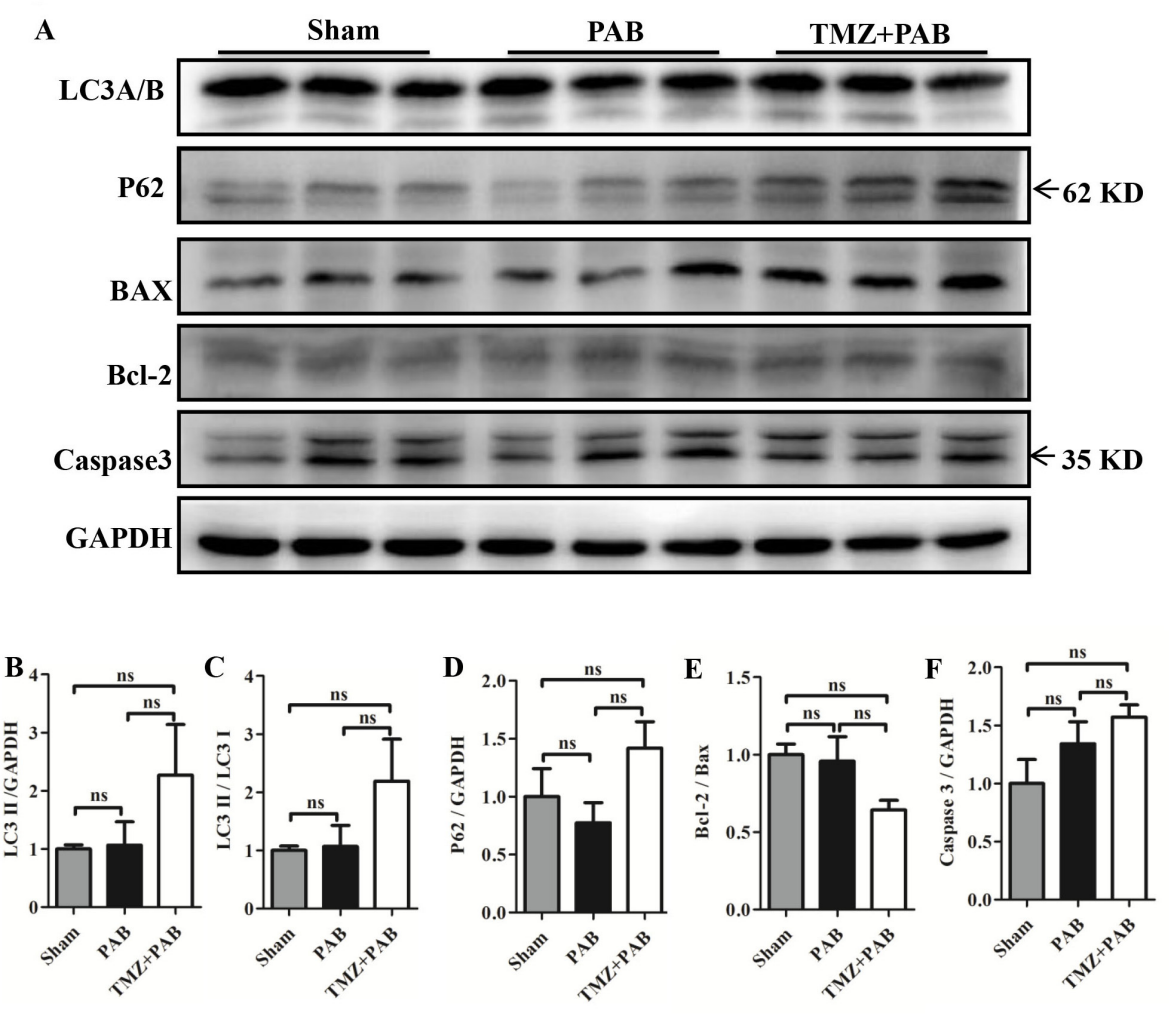
Detection of autophagy and apoptosis pathway by Western blot in each group **(A)** Representative WB images. **(B-F)** Statistical analysis of (A). Abbreviations: PAB, pulmonary artery banding; TMZ, trimedazidine; LC3, microtubule-associated protein 1 light chain 3; P62, ubiquitin-binding protein 62; BAX, bcl-2 associated X protein; Bcl-2, B-cell lymphoma 2;caspase-3, cysteine-aspartic proteases 3; GAPDH, glyceraldehyde 3-phosphate dehydrogenase. ^*^P < 0.05, ^**^P < 0.01, ^***^ *P*< 0.001, n = 4-8 in each group.

Figures 6A-6F show no significant differences in LC3 II, p62, Bcl-2/Bax, and caspase-3 between the PAB group and the sham groups, and TMZ treatment also had no effects.

## DISCUSSION

Acute RHF has a high mortality but receives little attention from clinicians and basic scientists [25, 26]. Both effective treatment and the molecular mechanisms of RHF are still obscured [27]. In this study, we successfully developed an RHF model in rats and confirmed the protective effects of TMZ in RHF.

### Acute right heart failure model

Experimental animal models for RHF, such as monocrotaline (MCT), PAB, and pulmonary lobectomy-induced models take a long time to develop right heart hypertrophy, and then RHF [28–32]. In addition, the MCT model always yields systemic adverse effects that are important confounding factors in the study of RHF [28]. Pulmonary lobectomy not only disrupts right heart function but also disrupts lung function [31]. PAB allows study of the right heart without comorbidities [32]. However, previous experimental PAB models utilized the same gauge regardless of variation in pulmonary artery diameter and did not mimic acute RHF characterized by rapidly increased, and then decreased, right ventricular pressure (RVP) and decreased blood pressure (BP) [33–35]. We analyzed data from rats and found that pulmonary artery diameter correlates with body weight and heart weight, which means a fixed gauge cannot develop a fixed constriction degree in a PAB model [36–38] (Supplementary Figure 2). We measured the pulmonary artery before PAB and selected a corresponding gauge according to the pulmonary artery diameter in each rat and developed a nearly identical constriction degree. Furthermore, we tried different constriction degrees and found rats with approximately 75% constriction degree of the pulmonary artery presented hemodynamic changes in acute RHF (transient soaring, and then lower RVP with decreased BP). The pathological markers BNP and βMHC increased, and αMHC decreased in PAB rats but not in the sham group (Figure 1). The acute RHF in rats was well developed.

### Effects of TMZ on acute right heart dysfunction

In the normal RV, coronary perfusion mainly occurs during systole, which means it is blocked by increased afterload caused by PH or pulmonary embolism [12, 14]. Hence, a chronic oxygen demand–supply mismatch might be an underlying mechanism in the transition to failure. Capillary density was reported to be reduced in several PH models, which supports the notion mentioned above [15–17]. Normally, the main substrate for the adult heart is fatty acids that metabolize to glucose and lactose under stress [39]. Fatty acid oxidation (FAO) is reduced in PH patients with severe hypertrophy [19], which appears to be an adaptive mechanism because TMZ alleviates PAB-induced chronic mild RV dysfunction [24]. TMZ also has beneficial effects in MTC-induced RVH [40]. In this study, in the acute RHF model, TMZ had protective effects in right heart function. TMZ treatment before PAB decreased BNP and βMHC levels and increased αMHC levels (Figure 1C). The hemodynamic parameters BP, HR, RVP, and dP/dT improved in the PAB+TMZ group compared with the PAB group (Figure 2). Echocardiographic evaluation 24 hours after operation indicated a higher ejection fraction of the RV in the PAB+TMZ group than in the PAB group (Figure 3). TMZ can alleviate PAB-induced acute RHF evaluated by right catheter and echocardiography.

### Underlying mechanism of TMZ improving acute right heart dysfunction

TMZ inhibits the long-chain fatty acid oxidation and increases glucose oxidation in patients with ischemic heart disease [23] and in patients with idiopathic dilated cardiomyopathy [41]. TMZ increases net O_2_ consumption, reduces formation of oxygen free radicals, and enhances cardiac mitochondrial function in MCT-induced RVH in rats [42]. Our study showed that PGC-1β and PPAR-α were decreased in the PAB-induced acute RHF model compared with the sham group. TMZ treatment before PAB increased not only PGC-1β and PPAR-α but also PGC-1α and PPAR-δ (Figure 4). PGC-1α and PGC-1β are expressed in brown fat, heart, kidney, skeletal muscle, and brain and induce the mitochondrial function [43, 44]. PGC-1 is reduced in animal models of heart disease, accompanied by a switch of substrate use from fatty acids to glucose [45, 46], and PGC-1α is a potent regulator of gluconeogenesis via induction of phosphoenol pyruvate carboxykinase and glucose-6-phosphatase and increased tricarboxylic acid cycle flux [47–50]. PPARα and PPARδ also promote glucose metabolism, increase cardiac levels of endogenous antioxidants, promote mitochondrial biogenesis, and inhibit cardiomyocyte apoptosis, and their deficiency impairs cardiac function in animal models [51, 52]. In addition, the mRNA of PDK4 was also reported to be increased along with PGC-1α after exercise in muscle tissue [53, 54] and downregulated in cardiac tissue during heart failure [55]. In this study, we found PDK4 had no increase in the PAB-induced acute RHF model but increased by TMZ treatment, even though it was reported to be increased in both the PH and PAB models of adaptive RV remodeling [56]. Collectively, TMZ treatment alleviated acute right heart dysfunction, possibly by increasing some metabolic gene profiles associated with mitochondrial biogenesis and glucose oxidation.

Our previous study showed that high phosphorylated level of Akt was present in the threonine 308 site but not in the serine 473 site in RV compared with LV, and PRAS40, a promotor of Akt activity, was increased in its phosphorylated form but total PRAS40 was not increased in RV compared with LV [11]. In this study, we found that phosphorylated Akt at serine 473 site was increased accompanied by decreased total Akt in the PAB group (Figures 5A-5D). TMZ treatment reversed the changes in Akt and increased phosphorylated PRAS40, a downstream activator of Akt and promotor of mTORC1 activity (Figures 5A, 5H, and 5I) [57]. PRAS40 treatment was reported to improve the metabolic profile and prevent the development of diabetic cardiomyopathy in obese mice [58]. Additionally, a previous study showed PRAS40 preferentially promotes protective mTORC2 signaling in diseased myocardium, and PRAS40 phosphorylation stimulates physiological hypertrophy both *in vitro* and *in vivo* [59]. The beneficial effects of TMZ in rats with acute RHF were possibly induced by increased PRAS40 activity. However, we need further study to confirm that TMZ directly activates PRAS40.

Apoptosis and autophagy have been reported to be factors in the pathological process of RHF [60–63]. However, we have not found any changes among the sham, PAB, and PAB+TMZ groups (Figure 6). The possible reason is that our observations were too early.

### Limitations

The limitations of our study are as follows: (1) We did not detect reactive oxygen species (ROS) that possibly contribute to the pathophysiological process of PAB-induced RHF. (2) We have not observed longer effects of TMZ in RHF that might be useful for investigating the mechanisms of RHF induced by PAB. (3) Further study is needed to prove that TMZ directly activates PRAS40 signaling.

## MATERIALS AND METHODS

### Animal model

Male Sprague Dawley rats (200g-250g) were fed a standard diet. In the PAB+TMZ group, TMZ (3.58 mg/kg/d) was administered by gavage for three days before PAB. All experiments were conducted in accordance with the Guide for the Care and Use of Laboratory Animals published by the US National Institutes of Health (NIH publication no. 85-23, revised in 1996) and the regulations on mouse welfare and ethics of Gansu Provincial Hospital. All procedures were approved by the Ethics Committee of Gansu Provincial Hospital (Lanzhou, China).

### Right ventricular catheterization and pulmonary artery banding

Open-chest RV catheterization was performed under general anesthesia in all animals (sodium pentobarbital, 50 mg/kg, i.p.). Before the procedure, the rats were intubated (16-gauge Teflon tube) and attached to a mechanical ventilator (Micro-Ventilator, UNO, Zevenaar, the Netherlands; ventilator settings: breathing frequency, 80 breaths per minute; pressures, 9/0 cmH_2_O; inspiratory/expiratory ratio, 1:1). The right ventricle was approached via a middle thoracotomy to the second rib and the sternum retracted by use of a chest retractor. RV pressures were recorded by use of a high-fidelity catheter tip transducer (Mikro-Tip SPR671, Millar Instruments, Houston, TX). Analyses were performed when steady state was reached over an interval of at least 10 seconds and averaged.

After right ventricular catheterization was established, fine-tip 45° angled forceps were used to gently separate the thymus and fat tissue from the pulmonary artery trunk. After identification of the pulmonary artery trunk, a Vernier caliper was used to measure the diameter of pulmonary artery (Supplementary Figure 2F). A vascular clip was then used to clip the pulmonary artery at the main pulmonary artery level, placing a small piece of a selected gauge blunt needle parallel to the main pulmonary artery to yield a residual cavity the same diameter of the gauge in lengths of the short shaft of the ellipse. Two knots were tied around the pulmonary artery and vascular clip to prevent clip and gauge slippage. In sham control rats, the entire procedure is identical, except for the clip of the pulmonary artery. After 1 minute, rats were sacrificed and hearts were harvested for further use. If the heart function was to be assessed by echocardiography after 24 hours, the chest retractor was removed and the rib cage was closed by use of a 6.0 prolene suture with an interrupted suture pattern and the skin was closed using a 6.0 prolene suture with a continuous suture pattern. To assess the effects of different banding degrees on right heart function, we used different gauges to produce different constrictions of pulmonary arteries. The success rate for establishing an acute RHF model is 86.8% (data not show).

### Calculation of constriction degree of pulmonary artery

The pulmonary artery was oval shaped after banding and the diameter of the gauge was the same length as the short shaft of the elliptic pulmonary artery. We assumed that the circumference of the pulmonary artery did not change before and after banding. According to the diameter of the pulmonary artery before banding and the length of the short shaft of the pulmonary artery with oval shaped deformation after banding, we could obtain the area of the pulmonary artery before and after banding, and then the constriction degree, indicated as area stenosis degree of the pulmonary artery, was calculated according the formula, banding degree (%) = 1− (π[r−b]/[2+b]b)/r^2^ (r, radius of pulmonary artery; b, the length of the short shaft of the elliptic pulmonary artery after banding) (Supplementary Figure 3).

### Echocardiography of right heart

Rats were anesthetized with isoflurane and subjected to echocardiography as described by Brittain at al. [64] and Cheng et al. [65]. A Vevo 770 (Visual Sonics), equipped with a 30-MHz transducer, was used for noninvasive transthoracic echocardiography. Two-dimensional guided M-mode tracings were recorded. The parasternal long axis M mode view was used to obtain RV chamber dimension, ejection fraction, and RV wall thickness. Echocardiography was performed without knowledge of the animal group.

### Western blotting analysis

Heart lysates of rats were prepared in lysis buffer (20 mM Tris, 150 mM NaCl, 10% glycerol, 20 mM glycerophosphate, 1% NP40, 5 mM EDTA, 0.5 mM EGTA, 1 mM Na3VO4, 0.5 mM PMSF, 1 mM benzamidine, 1 mM DTT, 50 mM NaF, 4 μM leupeptin, pH = 8.0). Samples were resolved by 10% SDS-PAGE and transferred to PVDF membranes (Millipore). Membranes were blocked with 5% non-fat milk in TBST (50 mM Tris, 150 mM NaCl, 0.5 mM Tween-20, pH = 7.5), and then incubated with primary antibodies overnight. Antibodies used in this study were purchased from Cell Signaling Technology (CST; Danvers, MA, USA), Bioworld: total Akt (CST #4691), phospho-Akt (Ser308) (CST #4060), phospho-Akt (Thr473) (CST #13038), phospho-GSK3β (Ser9) (CST #5558), LC3A/B (CST #12741), PRAS40 (CST #2691), phospho-PRAS40 (Thr246) (CST #13175), PTEN (CST #9188), phospho-PTEN (Ser380) (CST #9551), Bax (CST2772s), Bcl2 (CST3498s), GAPDH (#AP0063), anti-rabbit IgG, (HRP-linked antibody) (CST #7074). Image J software (NIH) was used to perform densitometric analysis (http://rsb.info.nih.gov/ij/).

### Quantitative real-time PCR for metabolism relative genes and fetal genes

Total RNA was extracted from RVs by use of TRIzol reagent (Invitrogen, Carlsbad, CA), according to the manufacturer's protocol. One microgram of total RNA from each specimen was reverse transcribed to cDNA by use of SuperScript Reverse Transcriptase and random hexamers as primers (Invitrogen). Quantitative real-time PCR (qRT-PCR) was performed with an ABI StepOnePlus instrument (Applied Biosystems, Foster City, CA) utilizing a 1×ITaQ SYBR Green Supermix Kit (Bio-Bad, Reinach, Switzerland) and 300 nmol/L as forward and reverse primers in a total volume of 20 μL. The mRNA level was based on the critical threshold (Ct) value. Primer sequences for quantitative real-time PCR are showed in Table 1, and 18S-rRNA was used as internal control.

**Table 1:** Primer sequences of metabolism genes in rat

Primer	Forward	Reverse
GLUT1	GCTGTGGCTGGCTTCTCTAA	CCGGAAGCGATCTCATCGAA
GLUT4	GGCAATGCCAAATTGCTCCA	GGCACAGTTAAGGTCCCCTC
PDK4	ACAATTCACGGAATGCCCCT	TACTTGGCGTAGAGACGGGA
PPARα	GAGTAGCCTGGGCTGCTTTT	CTGATCACCAGCAGAGGTCC
PPARγ	TACCACGGTTGATTTCTC	TCTACTTTGATCGCACTTT
PPARδ	CATCCGTTCTCTACCCAGCC	AATTCTGAGCCCGGAGTTGG
PGC-1α	TGGAGTGACATAGAGTGTGCTG	TATGTTCGCGGGCTCATTGT
PGC-1β	GGCCACACCTGTCTATGCTT	AGGCTTGTTGACATCCCGTT
ANP	GAGCAAATCCCGTATACAGTGC	ATCTTCTACCGGCATCTTCTCC
BNP	GCTGCTGGAGCTGATAAGAGAA	GTTCTTTTGTAGGGCCTTGGTC
α-MHC	ACATCAGTCAGCAGAACA	TTCCTCTAGCCTCTCACT
β-MHC	GCTGTTATTGCTGCCATT	TTATCATTCCGAACTGTC

Abbreviation: GLUT1, glucose transporter 1; GLUT4, glucose transporter 4; PPARα, peroxisome proliferator-activated receptor α; PPARγ, peroxisome proliferator-activated receptor γ; PPARδ, peroxisome proliferator-activated receptor δ; PGC-1α, peroxisome proliferator-activated receptor gamma coactivator 1α; PDK4, Pyruvate dehydrogenase lipoamide kinase isozyme.

### Statistics

Results of calculations were presented as means ± SEM. Differences in means between two groups were evaluated by application of unpaired two-tailed Student's *t* tests and those among multiple groups with one-way ANOVA followed by Bonferroni post hoc tests. Repeated-measures ANOVA were used to measure blood pressure, heart rate, and right ventricular pressure at multiple time points. All statistics was performed by GraphPad Prism 4.0 software (GraphPad, San Diego, CA, USA). *P* values of <0.05 were considered statistically significant.

## CONCLUSIONS

We successfully developed an acute RHF model by PAB at approximately 75% constriction degree. TMZ, an accelerator of energy metabolism, can alleviate PAB-induced acute RHF, possibly by increasing PGC-1α, PGC-1β, PPAR-α, PPAR-δ and PDK4 levels as well as increasing PRAS40 activity. Therefore, TMZ might be an alternative drug for patients with acute RHF. Also, these findings might provide new insights for therapeutic strategies that utilize PRAS40 directed against afterload-induced acute RHF.

## SUPPLEMENTARY MATERIALS FIGURES


