# Supplementary material for: Trimedazidine alleviates pulmonary artery banding-induced acute right heart dysfunction and activates PRAS40 in rats
Source: Oncotarget. 2017 Sep 8;8(54):92064–78. doi: 10.18632/oncotarget.20752 (PMC5696164; doi:10.18632/oncotarget.20752)
Supplement: Supplementary file 1 [file oncotarget-08-92064-s001.pdf]

Trimedazidine alleviates pulmonary artery banding-induced acute right heart dysfunction and activates PRAS40 in rats

SUPPLEMENTARY MATERIALS

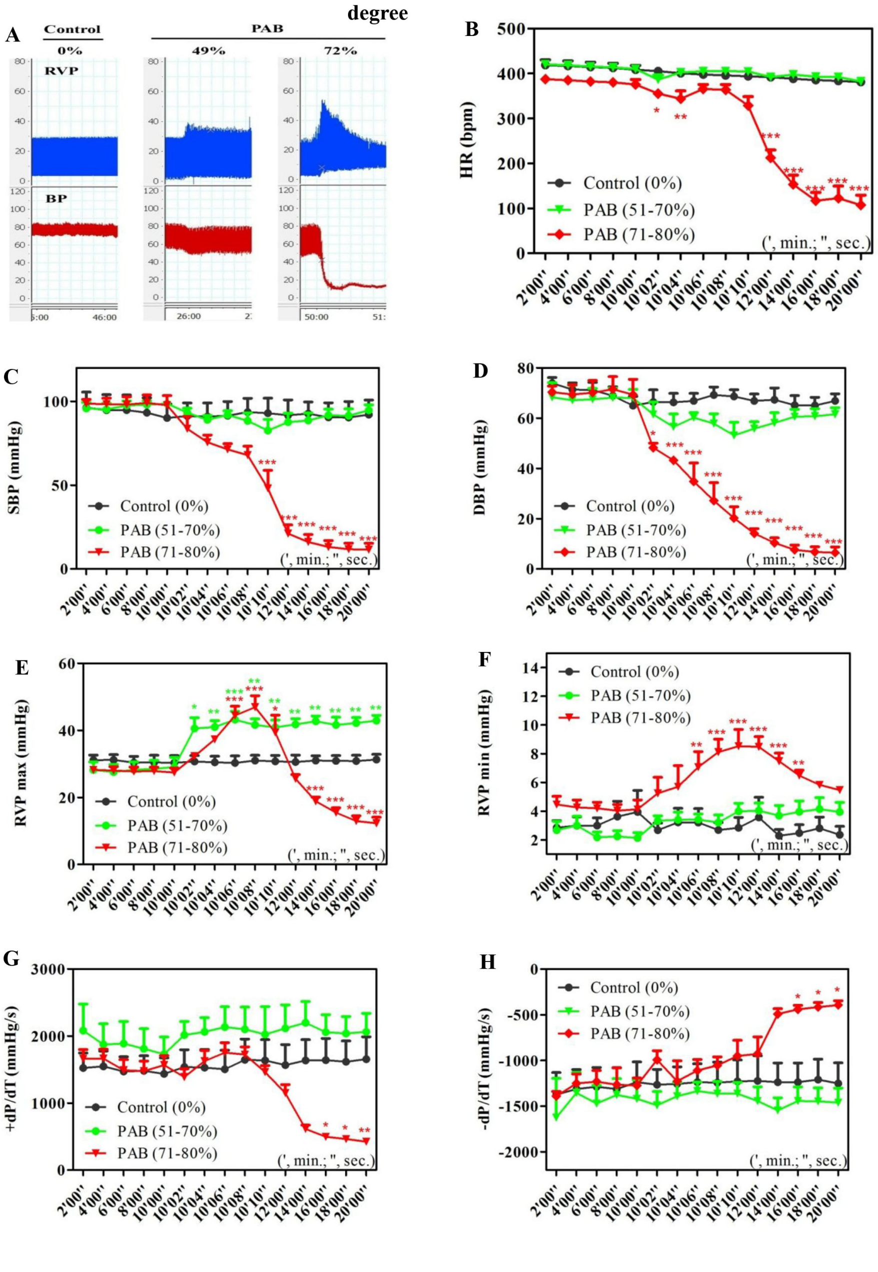

Supplementary Figure 1: Hemodynamic parameters under different pulmonary artery banding constriction degree. **(A)** Representative hemodynamic graphs. **(B)** Heart rate (HR) with time (mins) under constriction degrees of 51%-70% and 71%-80%. **(C)** and **(D)** Systolic blood pressure (SBP) and diastolic blood pressure (DBP) with time (mins) under constriction degrees 51%-70% and 71%-80%. **(E)** and **(F)** Maximum right ventricular pressure (RVP max) and minimum right ventricular pressure (RVP min) with time (min) under constriction degrees of 51%-70% and 71%-80%; **(G)** and **(H)** +dP/dT and –dP/dT with time (min) under constriction degrees of 51%-70% and 71%-80%. Banding time at 10 seconds; PAB, pulmonary artery banding; mins, minutes. $^{*}P < 0.05$, $^{**}P < 0.01$, $^{***}P < 0.001$, compared with controls; n = 4-8 in each group.

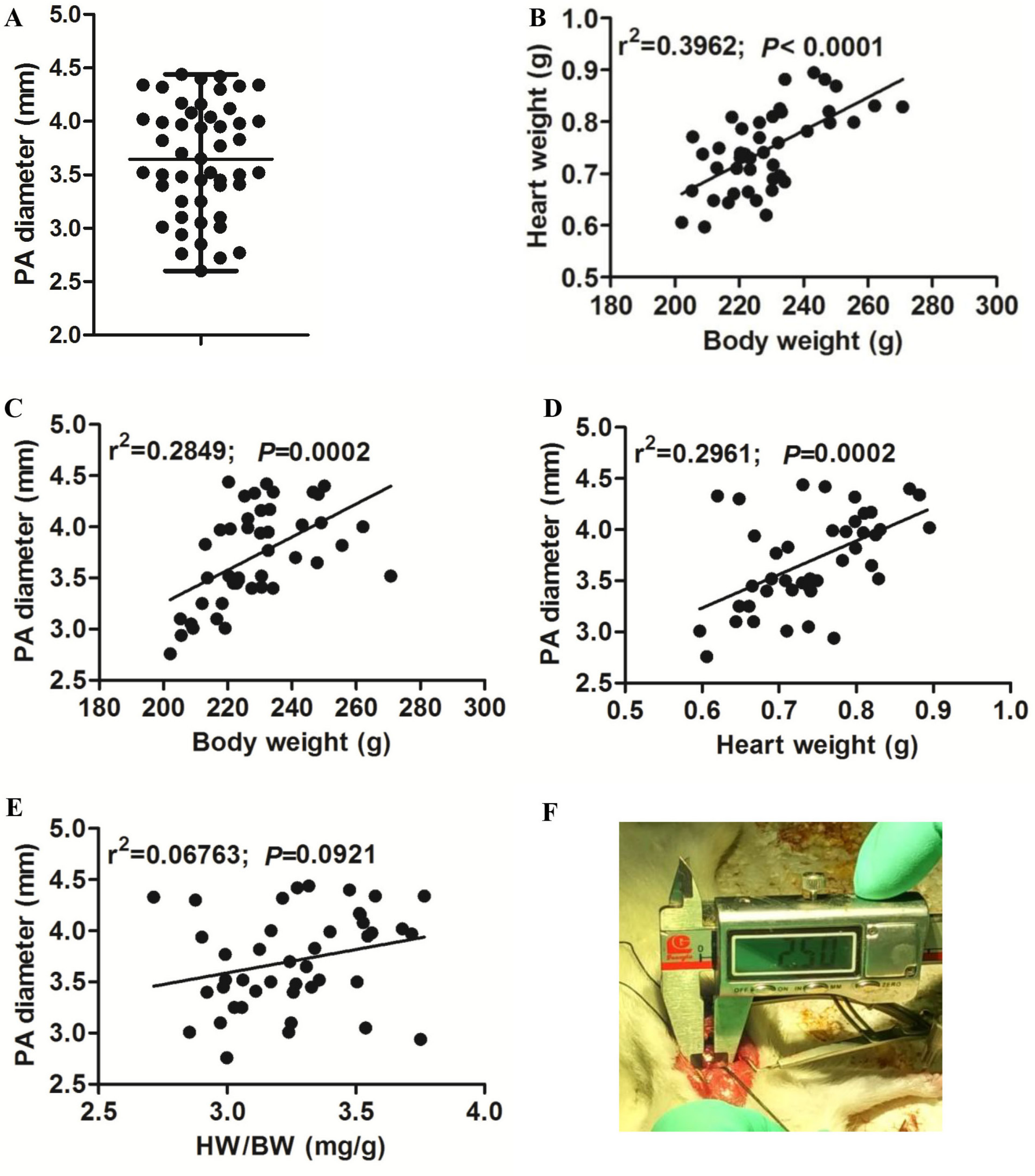

Supplementary Figure 2: Pulmonary artery diameter is variable and closely related to body weight and heart weight but not to the ratio of heart weight to body weight. **(A)** Statistical analysis of pulmonary artery diameter. **(B)** Relation of heart weight and body weight. **(C)** Relation of pulmonary artery diameter and heart weight. **(D)** Relation of pulmonary artery diameter and body weight. **(E)** Relation of pulmonary artery diameter and HW/BW. **(F)** Measurement of pulmonary artery diameter by use of Vernier calipers. PA, pulmonary artery; HW, heart weight; BW, body weight.

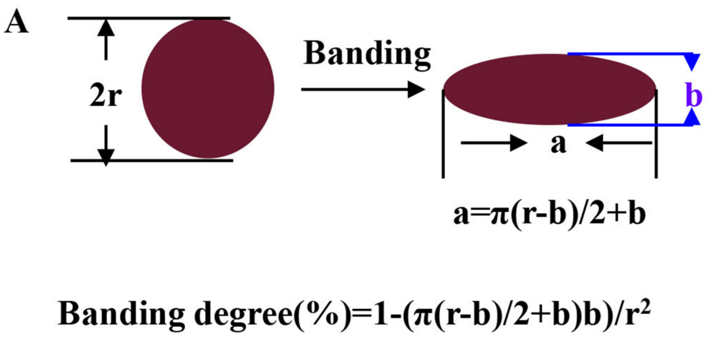

Supplementary Figure 3: Formula to calculate banding area of pulmonary artery. **(A)** Schematic diagram of pulmonary banding and formula for calculating banding area.
